# ^68^Ga-FAPI PET/CT for non-invasive characterization and activity assessment of ulcerative colitis and Crohn´s disease

**DOI:** 10.1007/s00259-025-07686-1

**Published:** 2026-01-02

**Authors:** Josefin Debus, Isabelle von Götze, Johannes Brandt, Robert Ehehalt, Anna-Maria Spektor, Philipp Mildenberger, Annika Gauss, Matthias Lang, Frederik M. Glatting, Mathias Schreckenberger, Rahul Kalla, Uwe Haberkorn, Manuel Röhrich

**Affiliations:** 1 Department of Nuclear Medicine, University Hospital Heidelberg, Heidelberg, Germany; 2 Department of Nuclear Medicine, University Hospital Mainz, Mainz, Germany; 3 Gastroenterology Outpatient Clinic Heidelberg, Heidelberg, Germany; 4 Institute of Medical Biostatistics, Epidemiology and Informatics, University Medical Centerof the Johannes Gutenberg University, 55131 Mainz, Germany; 5 Department of Gastroenterology, Hepatology and Infectious Diseases, University Hospital Heidelberg, Heidelberg, Germany; 6 Department of General, Visceral, and Thoracic Surgery, University Hospital Heidelberg, Heidelberg, Germany; 7 Endokrinologikum Wiesbaden, Wiesbaden, Germany; 8 Department of Radiation Oncology, University Medical Center Mannheim, Medical Faculty Mannheim, University of Heidelberg, Mannheim, Germany; 9 DKFZ-Hector Cancer Institute at the University Medical Center Mannheim, Mannheim, Germany; 10 Centre for Inflammation Research, Insititute for Regeneration and Repair, University of Edinburgh, Edinburgh, UK; 11 German Cancer Research Center (DKFZ), Heidelberg, Germany

**Keywords:** FAPI, Fibroblasts, Crohn´s disease, Ulcerative colitis, IBD disease activity

## Abstract

**Purpose:**

Inflammatory Bowel Diseases (IBD) comprise ulcerative colitis (UC) and Crohn’s disease (CD). Management of IBD requires assessment of disease activity, severity, extent and complications. Here, we describe the signal behavior of both CD and UC in ^68^Gallium- fibroblast activation protein inhibitor-based radiopharmaceuticals-46-positron emission tomography (^68^Ga-FAPI-46-PET) and evaluate the potential of ^68^Ga-FAPI-46-PET for activity assessment in IBD.

**Patients and methods:**

This analysis includes data of 43 IBD patients and 43 control patients examined by ^68^Ga-FAPI-46-PET/computed tomography (CT). Disease activity of IBD patients was assessed by colonoscopy. FAPI-positive gastrointestinal tract (GIT)-findings and healthy appearing GI structures were contoured. Non-IBD related FAPI-positive GIT-findings were ruled out by interdisciplinary consensus. Static and dynamic PET-parameters of FAPI-positive IBD lesions and healthy appearing GI structures were extracted and PET signalling was analyzed with respect to IBD subtype and disease activity.

**Results:**

We examined 20 CD patients and 23 UC patients (29 with active, 14 with inactive disease). FAPI-uptake in most healthy appearing GI structures of IBD patients was significantly increased compared to controls. Of 80 FAPI-positive GIT-findings, 14 were ruled out as non-IBD related and 66 FAPI-positive IBD lesions were analyzed. We observed equally high lesional FAPI-uptake in CD and UC. All patients with active disease showed at least one intensively FAPI-positive IBD lesion, while only 4/14 patients with inactive disease showed any FAPI-positive IBD lesion. Lesional and patientwise FAPI-uptake was significantly higher in active than in inactive disease. FAPI-positive IBD lesions showed a characteristic kinetic behaviour with two types of uptake patterns – one showing a continuous increase and the other an early peak followed by a plateau.

**Conclusion:**

^68^Ga-FAPI-46-PET/CT appears promising for assessing disease activity in terms of fibroblast activation in both CD and UC.

**Supplementary Information:**

The online version contains supplementary material available at 10.1007/s00259-025-07686-1.

## Introduction

Inflammatory Bowel Diseases (IBD), comprising ulcerative colitis (UC) and Crohn’s disease (CD), represent a complex spectrum of chronic inflammatory conditions of the gastrointestinal tract (GIT). IBD are considered to be a result of a dysregulated immune response arising from an interaction between genetic susceptibility and environmental factors such as diet and intestinal microbiome [1, 2]. Intestinal fibrosis is a crucial process in IBD pathophysiology, which is driven by fibroblasts that promote inflammation and fibrosis and could be used as targets to treat fibrosis [3, 4].

Effective management and monitoring of IBD require various diagnostic modalities to assess disease activity, severity, extent and associated complications. Colonoscopy and histopathological evaluation of mucosal biopsies, ultrasound, magnetic resonance imaging (MRI) and computed tomography (CT) enterography as well as laboratory markers (fecal calprotectin) are clinically established methods for diagnosis and monitoring of IBD [5]. However, these diagnostic modalities have limitations: Evaluation via colonoscopy is limited to the distal terminal ileum and at a mucosal level only; this is further limited in those with strictures of the bowel. Furthermore, colonoscopy carries risk of perforation and bleeding, especially in severe disease. Additionally, the assessment depends on the investigator resulting in an interobserver variability. [6, 7]. MRI and CT enterography only provide morphological information and are limited by a low spatial resolution and a low sensitivity for superficial mucosal lesions [8]. Positron emission tomography (PET) with ^18^Fluor-Fluorodeoxyglucose (^18^F-FDG) allows visualizing inflammation in IBD and has shown promising results in terms of supporting IBD diagnosis and depicting areas of active disease [9, 10]. However, physiological glucose uptake in the digestive tract, coexisting conditions such as diabetes and certain medications (e.g. metformine or corticosteroids) can influence ^18^F-FDG-uptake of the bowel, which limits the specificity of ^18^F-FDG-PET for IBD assessment [9, 11, 12].

^68^Gallium labelled (^68^Ga-) PET tracers targeting fibroblast activation protein (FAP) with FAP inhibitor-based radiopharmaceuticals (FAPIs) [13, 14] have been introduced with a focus on malignant conditions [15]. However, ^68^Ga-FAPI-PET has also proven valuable in non-malignant settings including chronic inflammation and various rheumatic and fibrotic diseases [16–18]. In a case report, Luo et al. have described FAPI-positivity of CD, but not UC [19]. In a recent study, Chen et al. [20] have shown the potential of ^68^Ga-FAPI-PET/CT for activity assessment and monitoring of CD.

Here, we describe the FAPI-uptake of both CD and UC IBD lesions and evaluate the potential of FAPI-PET for activity assessment in both IBD subtypes.

## Patients and methods

### Patients

From July 2023 to September 2024, 43 consecutive IBD patients were examined by ^68^Ga-FAPI-46-PET/CT. All patients were individually referred by their treating physicians from two outpatient facilities specialized in the treatment of IBD patients in Heidelberg, Germany. All patients had a histopathologic proven diagnosis of either Crohn’s disease or ulcerative colitis and were aged ≥ 18 years. Patients with diagnosed acute intestinal infection were not referred. 43 age- and sex-matched control patients without any known gastrointestinal disease or without tumor manifestations in GIT served as controls. Oncological patients of the control group had a low tumor burden. IBD Patients and control patients were included irrespective of their previous or current medication. Detailed information on the control cohort is given in supplemental Table 1. Written informed consent was obtained from all patients on an individual-patient basis following the regulations of the German Pharmaceuticals Act §13(2b). Retrospective analysis of imaging, clinical and pathological data was approved by the local institutional review board (study number S-115/2020).

**Table 1:** Basic clinical information on patients with inflammatory bowel disease (IBD) (Crohn´s disease (CD) or ulcerative colitis (UC)) and control group

	**IBD patients**	**Control group**
	43 consecutive patients with IBD (20 CD, 23 UC)	43 patients without IBD or gastrointestinal disease
**Age (y/o)**	18–75 (median 39)	5–71 (median 41),
**Gender**	27 male, 16 female	age & gender-matched
**IBD diagnosis**	ileocolonoscopy, histology, disease history, laboratory markers and ultrasound	

### Colonoscopy-based assessment of disease activity

Disease activity was assessed by colonoscopy prior to ^68^Ga-FAPI-46-PET/CT of all patients except one. In this patient, colonoscopy was delayed to 15 months after ^68^Ga-FAPI-46-PET/CT due to the patient´s wish. For the other patients, between colonoscopy and ^68^Ga-FAPI-46-PET/CT there was an average time span of 24.4 (± 11.8) days. Two board approved gastroenterologists determined endoscopic disease activity in UC patients using the Mayo Endoscopic Score (MES) [21] and in CD patients based on the presence of ulcers. MES of 0–1 were classified as endoscopic remission, while MES of 2–3 indicated active disease. The presence of ulcers in CD signified active disease [22].

### ^68^GA-FAPI-46-PET/CT

FAPI-46 was synthesized and labelled with ^68^Ga according to established protocols [10]. PET-scans were obtained on a Biograph mCT Flow PET/CT scanner (Siemens Medical Solutions). To correct for photon attenuation in tissue, a low-dose CT scan (130 kiloelectron volt (keV), 30 milliampere-seconds (mAs)) was performed using CARE Dose4D with 5 millimeter (mm) slice thickness, 3–4 mm increment, and a soft-tissue kernel. Immediately after intravenous administration of the tracer (mean activity: 238.7 megabecquerel (MBq), range: 170–295 MBq), dynamic PET scans of the abdomen were performed over 60 minutes (min) to characterize lesional uptake kinetics as we have observed differential FAPI-uptake kinetics of different relevant pathologies such as cancer, premalignant lesions, fibrosis and inflammation in our previous work [17, 23, 24]. Directly after dynamic PET scans, whole body PET was acquired over 30 min. Data were corrected for random events, scatter, and half-life, and reconstructed with an expectation–maximization algorithm (2 iterations, 21 subsets) and Gaussian filtering. In two patients, only static whole-body PET/CT scans were performed due to logistical problems. One patient (with inactive disease) only received a dynamic abdominal PET/CT scan due to a technical problem of the PET/CT scanner.

### Image analysis

All image analyses were performed using Pmod software (version 4.2). Healthy appearing regions of the gastrointestinal tract (floor of mouth, esophagus, stomach, duodenum, jejunum, ileum, ascending colon, transverse colon, descending colon, sigmoid colon and rectum) were contoured in IBD and control patients [25]. FAPI-positive GIT findings were contoured using an isocontour (20% to 70%) in which the readers (JD and MR) were blinded for the endoscopic findings. All FAPI-positive GIT findings were reviewed by an experienced board certified gastroenterologist (RE, > 20 years) and an experienced board certified nuclear medicine physician (MR, > 5 years) to identify non-IBD related FAPI-positive findings and exclude these from further analysis. For static imaging, the mean and maximum standardized uptake values (SUVmean, SUVmax) of all FAPI-positive IBD lesions and healthy appearing GI structures were extracted. In addition, FAPI-volume per patient was calculated by multiplication of SUVmean and “volume of interest” (VOI)-volume of each FAPI-positive IBD lesion. Differences between FAPI-uptake of healthy appearing GI structures in IBD patients versus control patients, between CD and UC and between inactive and active disease were analyzed. Dynamic FAPI-PET data was used for extraction of time activity curves (TACs) and calculation of time to peak (TTP) of FAPI-positive IBD lesions and healthy appearing GI structures.

### Statistical analysis

For all statistical analyses, GraphPad PRISM (version 10) was used. Patient demographics, disease history and PET/CT parameters were analyzed using descriptive analyses. For static and dynamic PET-parameters, a normality and lognormality test was performed and according to the result either an unpaired t-test or Mann–Whitney test was done. Local p values of less than 0.05 were considered statistically significant. Given the exploratory character of this analysis, no adjustment for multiple testing was performed.

## Results

### Patients, diagnoses and disease activity

43 patients (16 female, 27 male) were examined by ^68^Ga-FAPI-46-PET/CT (aged 18 to 75 years, median 39 years). The median time between initial IBD diagnosis and PET/CT imaging was 13.5 years (range 1 to 45 years). Basic clinical information on the IBD patients and the control cohort is displayed in Table 1 and detailed clinical patient characteristics including their medication are provided in supplemental Table 2. Twenty patients were diagnosed with CD, 23 with UC. On the basis of colonoscopy-based classification, 29 patients had active disease and 14 inactive disease after standard of care treatment. 13/14 patients with inactive disease were still on medication according to the European medical guidelines during the time of clinical examinations (see supplemental Table 2).

### Healthy appearing gastrointestinal tract in IBD and control patients

**Fig. 1:**
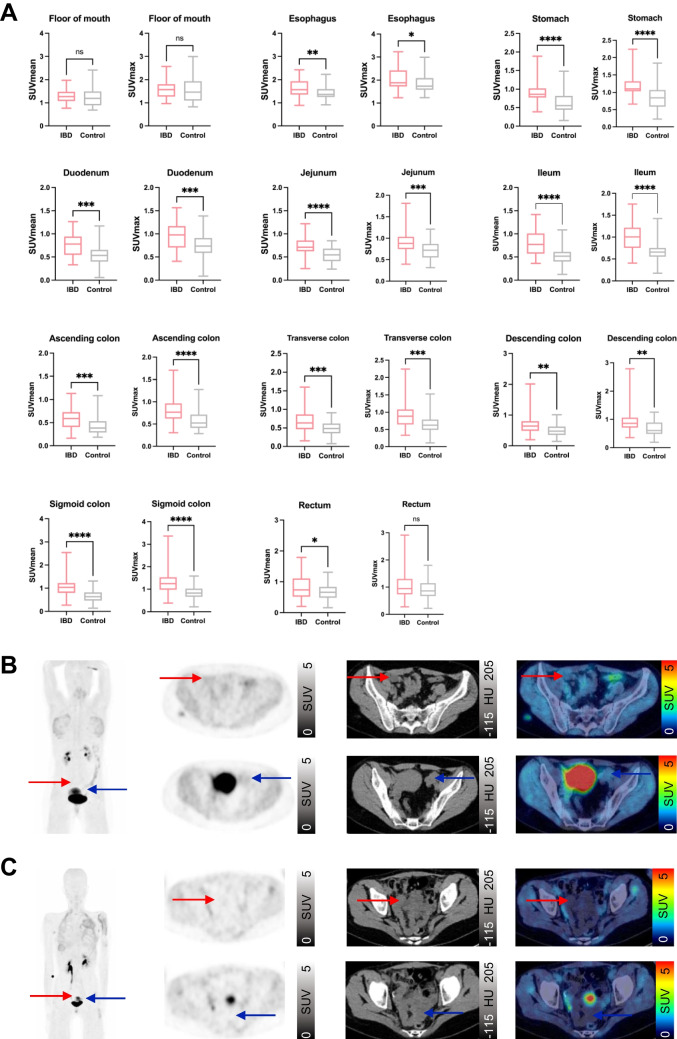
Static ^68^Gallium- fibroblast activation protein inhibitor-based radiopharmaceuticals-46-positron emission tomography (^68^Ga-FAPI-46-PET) imaging of healthy appearing gastrointestinal (GI) structures in 42 patients with inflammatory bowel disease (IBD) and 42 control patients. A SUVmean and SUVmax of healthy appearing GI structures in IBD and control patients. Boxplots represent interquartile ranges, whiskers represent minimum and maximum values. Horizontal lines within boxes indicate medians. Stars indicate significant differences: * = < 0.05, ** = *p < *0.01, *** = *p < *0.001, **** = *p < *0.0001, ns = not significant. B,C Two examples of static ^68^Ga-FAPI-46-PET imaging of a 38- year old female with IBD (Crohn’s disease (CD)) (B) and a 17-year old female control patient (C). From left to right, maximum intensity projection (MIP), transversal PET, transversal computed tomography (CT) and fused PET/CT are shown. Red arrow indicates location of ileum and blue arrow indicates location of sigmoid colon

There was a significantly higher FAPI-uptake in radiologically healthy appearing regions of the gastrointestinal tract of IBD patients compared to the control group. Particularly stomach (SUVmean and max), jejunum (SUVmean), ileum (SUVmean and max), ascending colon (SUVmax) and sigmoid colon (SUVmean and max) showed a highly significant difference (*p < *0.0001). There were no significant differences between IBD cases and controls between floor of the mouth and rectum (SUVmean and SUVmax) and rectum (SUVmax) (Fig. 1).

### FAPI-positive GIT findings in IBD patients

Of 80 FAPI-positive GIT-findings, 14 were excluded from further analysis because these findings were considered consequences from previous interventions or medical conditions after consensus read: enema (9), surgery (2), hemorrhoids (2) and diverticulitis (1). In total, 66 FAPI-positive GIT findings were considered FAPI-positive IBD lesions and included into further analysis (see supplemental Table 3).

### FAPI-uptake of FAPI-positive IBD lesions in CD and UC

**Fig. 2:**
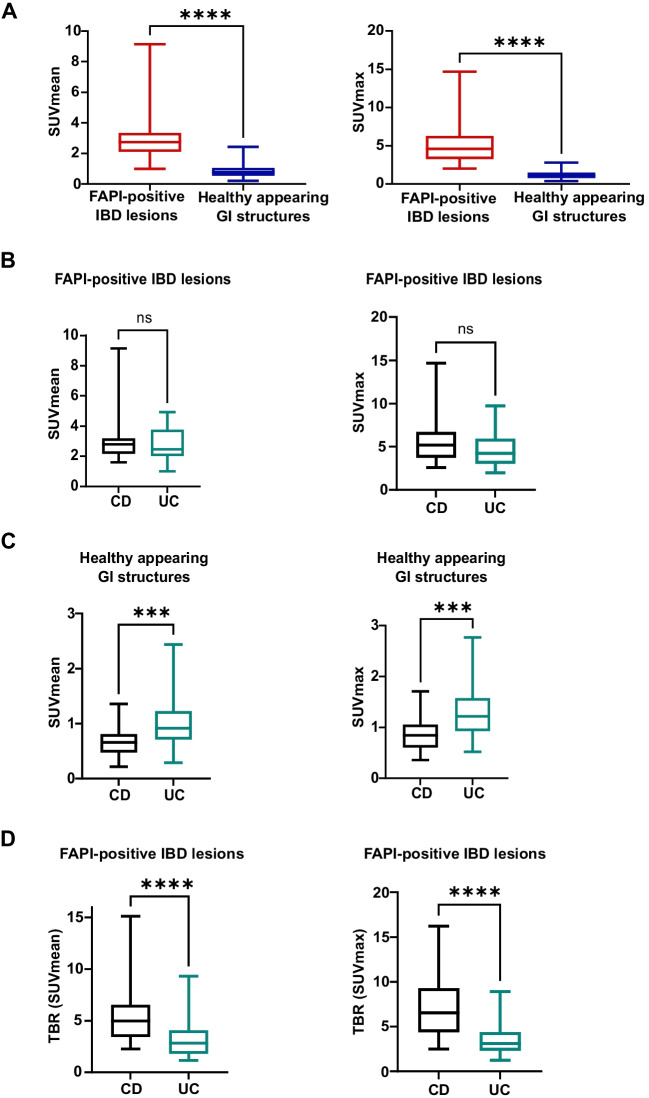
Static ^68^Gallium- fibroblast activation protein inhibitor-based radiopharmaceuticals-46-positron emission tomography (^68^Ga-FAPI-46-PET) imaging of 42 patients with Crohn´s disease (CD) or ulcerative colitis (UC). A SUVmean and SUVmax of FAPI-positive inflammatory bowel disease (IBD) lesions and healthy appearing gastrointestinal (GI) structures. Boxplots represent interquartile ranges, whiskers represent minimum and maximum value. Horizontal line within box indicates median. Stars indicate significant differences: **** = *p < *0.0001, ns = not significant. B Boxplots of SUVmean and SUVmax of FAPI-positive IBD lesions in CD and UC. Boxes represent interquartile ranges, whiskers represent minimum and maximum value. Horizontal lines within boxes indicate medians. Stars indicate significant differences: ns = not significant. C Boxplots of SUVmean and SUVmax of healthy appearing GI structures. Stars indicate significant differences: *** = *p < *0.001. D Boxplots of corresponding target to-background ratios (TBR) of FAPI-positive IBD lesions. Contralateral healthy appearing GI structures act as background. Boxes represent interquartile ranges, whiskers represent minimum and maximum values. Horizontal line within boxes indicates medians. Stars indicate significant differences: **** = *p < *0.0001

**Fig. 3:**
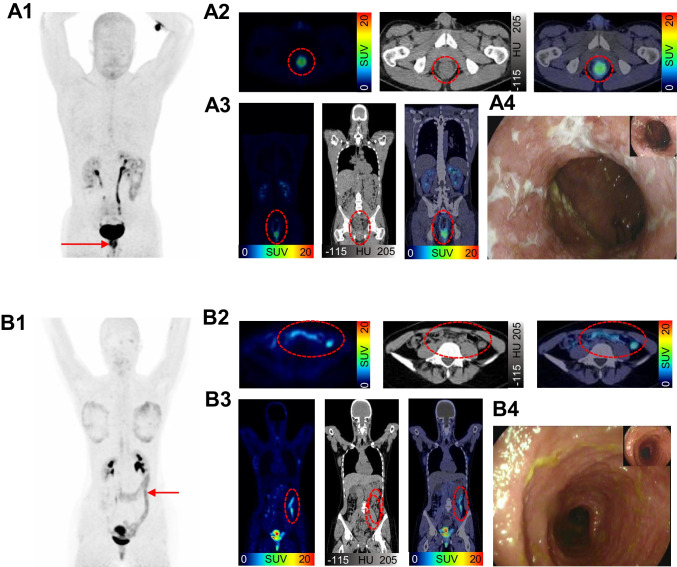
Examples of static ^68^Gallium- fibroblast activation protein inhibitor-based radiopharmaceuticals-46-positron emission tomography/computed tomography (^68^Ga-FAPI-46-PET/CT) imaging of 32-year old male with Crohn´s disease (CD) (A) and of 18-year old female with ulcerative colitis (UC) (B). 1 ^68^Ga-FAPI-46-PET with maximum intensity projection (MIP) 2 Transversal ^68^Ga-FAPI-46-PET/CT and fused ^68^Ga-FAPI-46-PET/CT 3 Coronal ^68^Ga-FAPI-46-PET/CT and fused ^68^Ga-FAPI-46-PET/CT 4 Example of colonoscopy. The FAPI-positive inflammatory bowel disease (IBD) lesion of CD in the rectum (see arrow in A1) is seen with a high uptake (SUVmax: 9.3) in reconstructed images (see red circle in A2 and A3) and corresponds to the endoscopic finding in A4. The example in B1 shows a long-standing FAPI-positive IBD lesion (SUVmax: 6.8) in the colon in UC, which is also visible on reconstructed ^68^Ga-FAPI-46-PET/CT in B2 and B3. Also this finding corresponds to the endoscopic reading

The median SUVmean/max of all FAPI-positive IBD lesions was 2.7 (range 1.0 to 9.1) and 4.6 (range of 2.0 to 14.7) significantly exceeding the FAPI-uptake of healthy appearing GI structures, which showed a median SUVmean of 0.8 (range 0.2 to 2.4) and a median SUVmax of 1.0 with a range of 0.4 to 2.8. (Fig. 2A.). We observed equally high FAPI-46-uptake of FAPI-positive IBD lesions in the 20 CD patients (median SUVmean: 2.8 (range 1.6 to 9.1), median SUVmax: 5.2 (range 2.6 to 14.7)) and the 23 UC patients (median SUVmean: 2.5 (range 1.0 to 4.9), median SUVmax: 4.2 (range 2.0 to 9.7)) (Fig. 2B). However, a significantly increased FAPI-uptake of the healthy appearing GI structures of UC patients (median SUVmean: 0.9 (range 0.3 to 2.4; median SUVmax: 1.2 (range 0.5 to 2.8)) was detected compared to that of CD patients (median SUVmean: 0.7 (range 0.2 to 1.4); median SUVmax: 0.8 (range 0.4 to 1.7)) (Fig. 2C). This resulted in highly significantly (*p < *0.001) increased target to-background ratios (TBR) of FAPI-positive IBD lesions to contralateral healthy appearing GIT structures in CD (median TBR (SUVmean/max: 5.0/6.5) compared to FAPI-positive IBD lesions in UC (median TBR (SUVmean/max): 2.8/3.1) (Fig. 2D). Exemplary ^68^Ga-FAPI-46-PET/CT images and corresponding colonoscopy findings of a patient with CD and a patient with UC are shown in Fig. 3.

### FAPI-signalling in active and inactive disease

**Fig. 4:**
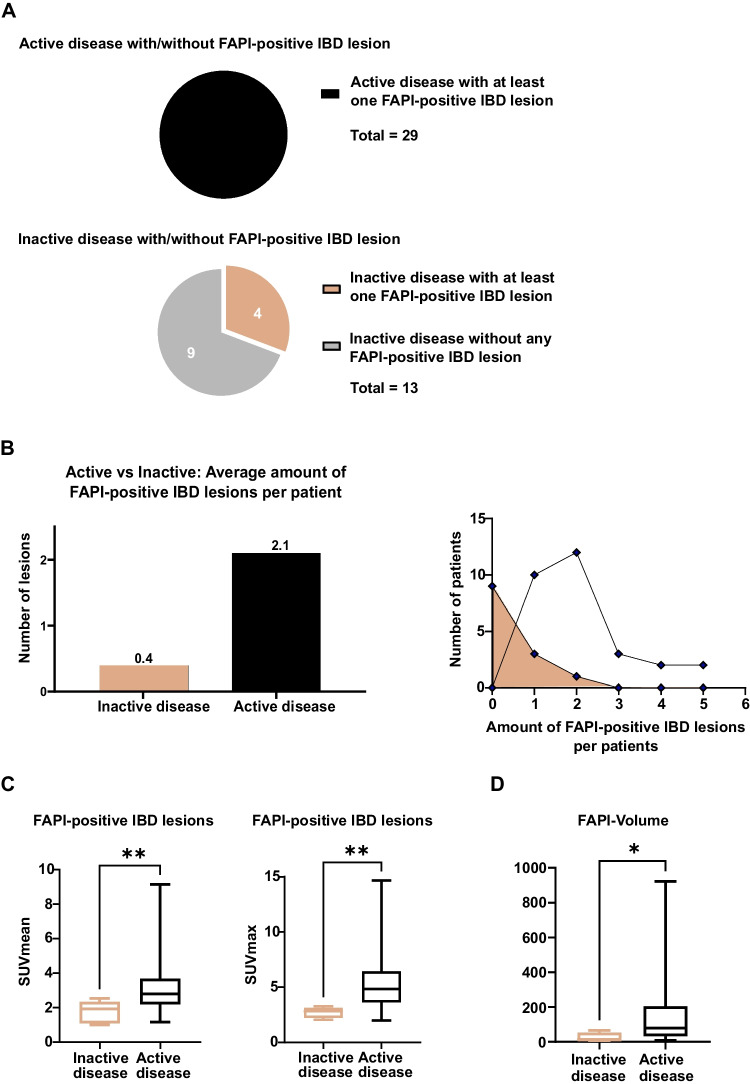
Quantitative analysis of static ^68^Gallium- fibroblast activation protein inhibitor-based radiopharmaceuticals-46-positron emission tomography/computed tomography (^68^Ga-FAPI-46-PET/CT) imaging of 42 patients with inactive and active inflammatory bowel disease (IBD). A Pie charts of patients with inactive or active IBD. B Bars and line graphs of patients with inactive or active IBD. Line graph represents the correlation between number of patients and FAPI-positive IBD lesions per patient. C Boxplots of SUVmean and SUVmax of FAPI-positive IBD lesions. Boxes represent interquartile ranges, whiskers represent minimum and maximum values. Horizontal line within box indicate medians. Stars indicate significant differences: ** = *p < *0.01. D Boxplots of median FAPI-volume per patient in patients with inactive or active disease. Boxes represent interquartile ranges, whiskers represent minimum and maximum values. Horizontal lines within box indicate medians. Stars indicate significant differences: * = < 0.05

**Fig. 5:**
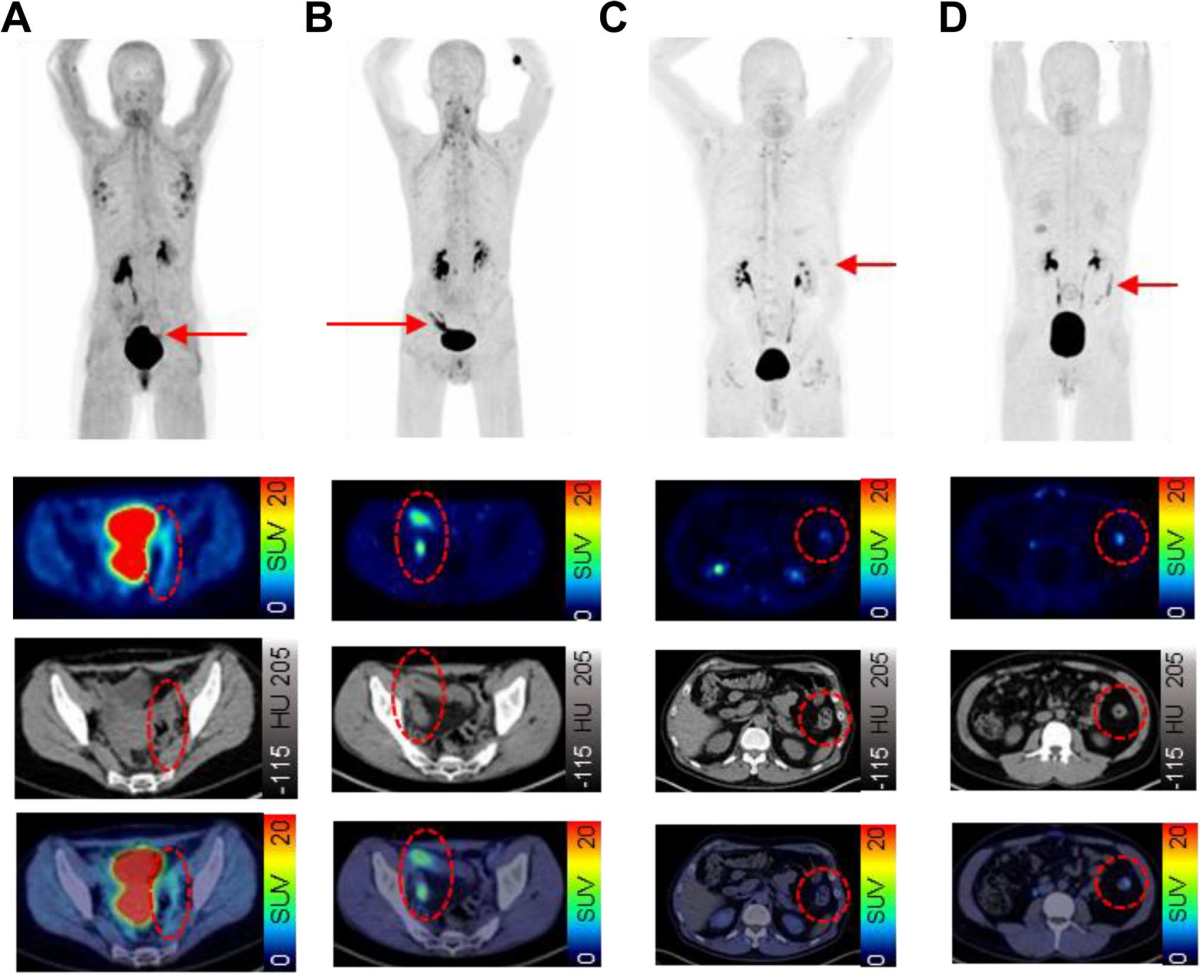
Exemplary cases of inactive and active Crohn´s disease (CD) and ulcerative colitis (UC) of static ^68^Gallium- fibroblast activation protein inhibitor-based radiopharmaceuticals-46-positron emission tomography/computed tomography (^68^Ga-FAPI-46-PET/CT) imaging. For each case, we show maximum intensity projection (MIP) (upper row) and transversal PET, CT and fused images (second, third and fourth row). FAPI-positive inflammatory bowel disease (IBD) lesions are indicated by arrows (MIP) and red circles (transversal images). **A** CD, inactive disease: 40-year old female with CD in remission. FAPI-positive IBD lesion (SUVmax: 3.3) in sigmoid colon. **B** CD, active disease: 38-year old male with active CD. FAPI-positive IBD lesion (SUVmax: 8.0) in ileum. **C** UC, inactive disease: 70-year old male with inactive UC. FAPI-positive IBD lesion (SUVmax: 2.8) in descending colon. **D** UC, active disease: 30-year old male with active UC. FAPI-positive IBD lesion (SUVmax:5.9) in descending colon

All 29 patients with active disease showed at least one intensively FAPI-positive IBD lesion. In contrary, among patients with inactive disease only 4/14 showed any FAPI-positive IBD lesions (Fig. 4A). In active disease, a mean of 2.1 FAPI-positive IBD lesions per patient was observed, in inactive disease 0.4 per patient (Fig. 4B). Concordantly, FAPI-positive IBD lesions of patients with active disease showed a higher FAPI-uptake in terms of median SUVmean/max than those of patients with inactive disease (Fig. 4C.). Furthermore, the median FAPI-volume per patient was significantly higher in patients with active disease than in patients with inactive disease (Fig. 4D). Exemplary images of CD and UC patients with active and inactive disease are shown in Fig. 5. Of note, in 7 patients with active disease, corticosteroid therapy has been initiated between colonoscopy and ^68^Ga-FAPI-46-PET/CT and 11 patients were under continuous corticosteroid therapy. All of them showed equally intense FAPI-positivity of their IBD lesions as patients without corticosteroid treatment (see supplemental Table 4).

### TTP and TAC characteristics and TTP of IBD lesions and healthy appearing GIT structures in dynamic FAPI-PET imaging

**Fig. 6:**
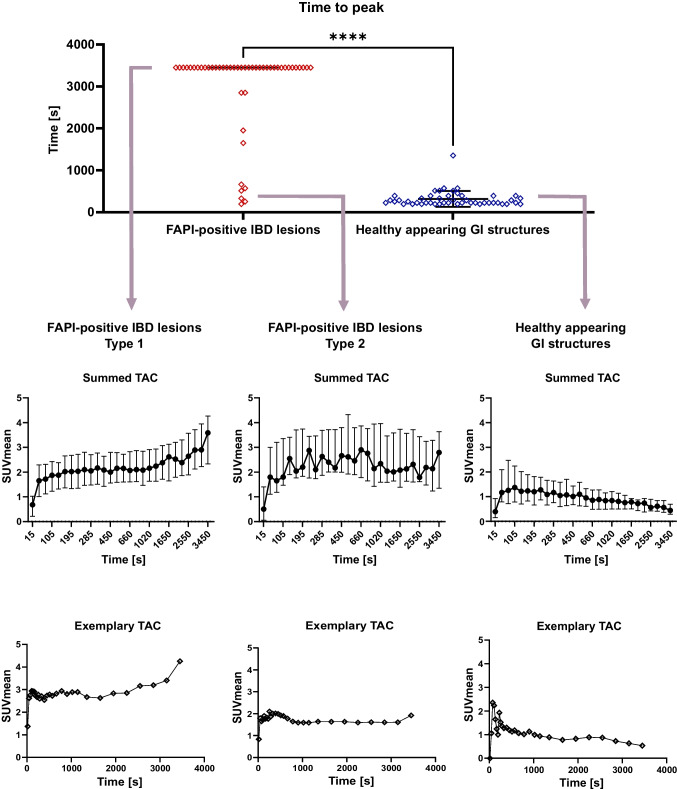
Dynamic ^68^Gallium- fibroblast activation protein inhibitor-based radiopharmaceuticals-46-positron emission tomography (^68^Ga-FAPI-46-PET) imaging properties of FAPI-positive inflammatory bowel disease (IBD) lesions and healthy appearing gastrointestinal (GI) structures of 41 patients. A Scatter dot plot of Time to peak (based on SUVmean) of FAPI-positive IBD lesions and healthy appearing GI structures. Horizontal line shows median. Whiskers show interquartile range. Time activity curves (TAC). Upper row shows averaged kinetic tracer uptake. Dot indicates median, whiskers indicate interquartile range. Lower row shows example for each type. Dot indicates mean, whiskers indicate standard deviation. Upper row shows averaged, lower row shows example. FAPI-positive IBD lesion Type 1 has its time to peak later than 1800s. FAPI-positive IBD lesion Type 2 has its time to peak earlier than 1725s

Dynamic PET/CT were performed and evaluated in 41 patients. 48 of the 66 FAPI-positive IBD lesions of patients with static imaging were covered by the corresponding dynamic field of view. The patient examined only by dynamic imaging had one FAPI-positive IBD lesion so that dynamic imaging of 49 FAPI-positive IBD lesions was analyzed. The median TTP of all FAPI-positive IBD lesions was highly significantly longer (*p < *0.001) than that of healthy appearing GI structures. Hereby, FAPI-positive IBD lesions of type 1 (42 lesions) had a late TTP (>1800 s, second half of dynamic acquisition), FAPI-positive IBD lesions of type 2 (7 lesions) an early TTP (< 1800 s, first half of acquisition). For healthy appearing GI structures, the summed time activity curve (TAC) reached an average peak at 105 s after injection followed by continuous decline to fifty percent of the peak activity. In FAPI-positive IBD lesions, we observed steep early increase similar to that in healthy appearing GI structures. After that increase, the two types of FAPI-positive lesions could be differentiated: FAPI-positive IBD lesions of type 1 had a continuously increasing TAC until the end of the measurement. FAPI-positive IBD lesions of type 2, showed an average peak at 660 s, which was followed by a nearly constant TAC (Fig. 6).

## Discussion

IBD, comprising CD and UC, present diagnostic and therapeutic challenges due to their heterogeneous manifestations and complex pathology. In this pilot study, we demonstrated the potential of ^68^Ga-FAPI-46-PET/CT as a non-invasive imaging modality in patients with IBD. Our findings revealed significantly higher FAPI-uptake in healthy appearing regions of the gastrointestinal tract in patients with IBD compared to control patients. These observations suggest a broader pathological process of subtle chronic inflammation and fibroblast activation beyond the endoscopically discernible disease [26]. Interestingly, healthy appearing colon showed a higher FAPI uptake in UC patients than in CD patients. This finding may reflect the diffuse nature of inflammation in UC compared to the localized, transmural inflammation typical of CD [27]. It would be of interest for future investigations if the signal of healthy appearing GI structures has a prognostic value for disease activity and progression of the disease burden of IBD.

While several studies have demonstrated the potential of FAPI-PET for assessing CD, our study represents, to our knowledge, the largest cohort including both ulcerative colitis and CD. We observed similar uptake patterns in both IBD subtypes, differing from the case report by Luo et al. [19], which described FAPI-positivity in CD but not UC. Chen et al. [20] also reported a correlation between FAPI uptake, inflammation, and disease activity in CD, with better lesion detection than CT enterography. Moreover, Scharitzer et al. [28] found that intestinal FAPI uptake correlated with histologically confirmed fibrosis in CD. Our findings align with these results and extend them to both main IBD subtypes, though sensitivity and specificity analyses were limited by sample size.

With respect to IBD activity assessment, we observed a broad overall concordance of endoscopically assessed disease activity and lesional as well as patientwise FAPI-positivity: Patients with endoscopically active IBD showed FAPI-positive IBD lesions with significantly higher FAPI-uptake and higher patientwise FAPI-volumes compared to those in endoscopically proven remission. This suggests a potential of ^68^Ga-FAPI-46-PET/CT in identifying areas of active disease in a non-invasive approach. However, 4 out of 14 patients with an endoscopically inactive disease had FAPI-positive IBD lesions in the bowel. The presence of FAPI-positive IBD lesions in endoscopically remitted patients makes it tempting to speculate that FAPI-46-PET may be even more sensitive for the detection of disease activity than colonoscopy. In our previous work on large vessel vasculitis (LVV), we also observed preserved FAPI-uptake in the vessel wall of clinically remitted LVV patients. This is in line with our findings in IBD and supports the concept of long-term fibroblast activation in chronic inflammatory diseases [29], potentially reflecting chronic inflammation or fibroblast involvement in wound healing processes. However, a consensus interpretation of the phenomenon of long-term fibroblast activation based on tissue-based biological data is not available to date. Dynamic PET/CT imaging enabled us to differentiate FAPI-positive IBD lesions into two subtypes based on their kinetic patterns of FAPI uptake (continuous increase versus early peak followed by a plateau). One possible biological explanation for the differential kinetic behaviour of IBD lesions may be differential underlying disease status with either predominantly fibrotic or inflammatory activity, which may be reflected by different patterns of FAPI-uptake over time. These differential uptake patterns need to be further assessed in synopsis with their pathological correlates and stages of the diseases in future studies.

Schmidkonz et al. [30] previously described the discrimination between inflammatory and fibrotic lesions in IgG4-related disease. The therapeutic relevance of this discrimination in strictures of IBD requires specific molecular imaging modalities. [16]. ^18^F-FDG-PET imaging has been tested in patients with IBD and showed promising results in detecting lesions. However, FAPI imaging might have a higher diagnostic performance compared to FDG-PET in chronic inflammatory diseases. While FAPI detects specific processes involving fibroblasts leading to fibrogenesis, FDG depicts cells metabolizing glucose especially in acute inflammation. Thus, the presence of physiological glucose uptake in the digestive tract might limit the TBR of FDG. [31, 32]. In addition, glucose uptake can be influenced by corticosteroids, a common medication in IBD. There is, however, no rationale to assume similar changes for FAPI-uptake. In our dataset FAPI-positivity of IBD patients with active disease and corticosteroid medication was not lower than in IBD patients without corticosteroid medication.

Taken together, non-invasive assessment of fibroblast activation by FAPI-PET provides a biologically based, clinically relevant perspective on IBD disease activity, complementing traditional established imaging modalities that concentrate on morphological or metabolic changes. However, it is essential to rule out false-positive findings to avoid misinterpretation of FAPI-positive GIT findings. This underlines the importance of interdisciplinary interpretation of imaging findings of IBD patients.

Despite these promising results, several limitations of this study must be considered. Firstly, the relatively small number of patients and retrospective design represent important limitations of our findings. Larger, multicenter studies are necessary to validate the actual diagnostic and prognostic value of FAPI-46-PET/CT in IBD. The heterogeneity of our cohort, including variations in treatment history and imaging intervals, may have influenced the results. A potential selection bias may exist due to the retrospective design of the study. Moreover, at least in single cases there was a certain time lag between colonoscopy and FAPI-46-PET/CT which might have caused a slight change in FAPI-uptake. This also applies to the control cohort which had different medication and comorbidities compared to the patients with IBD. Next, our control cohort consists of oncological patients. Although we have excluded patients with known GI diseases and tumor manifestations in the GIT activation of fibroblasts located in the GIT by inapparent micrometastases and consecutively enhanced FAPI-46 uptake cannot be definitely excluded in the control group.

Further limitations must be considered with respect to lesion characterization and statistical evaluation: Although exclusions for non-IBD related FAPI-positive findings were made by consensus, some subjectivity may persist. In addition, the absence of multiple testing correction inflates type 1 error risk and the observed associations should be interpreted with caution. Finally, an intraindividual comparison of FAPI-positive GIT findings, endoscopic and bioptic findings is not provided here. Such an analysis would allow a definitive characterization of FAPI-positive lesions as IBD or non IBD-related based on histology. However, this would exceed the scope of this manuscript. A lesionwise analysis of all FAPI-positive GIT findings and all available colonoscopy and biopsy results of this dataset is under way and will be published separately. Nevertheless, these findings on FAPI-PET for activity assessment of both IBD subtypes provide a rationale for further clinical investigations, e.g. longitudinal studies analyzing the predictive value of FAPI-based activity assessment of IBD and long-term outcomes.

## Conclusion

^68^Ga-FAPI-46-PET/CT is a promising imaging modality for assessing fibroblast activation in IBD. Prospective evaluation of its potential for disease monitoring in clinical trials is warranted.

## Supplementary Information

Below is the link to the electronic supplementary material.

Supplementary file1 (DOCX 51 KB)

## Data Availability

The data used and/or analyzed during the current study are available from the corresponding author upon reasonable request.^.^
