# Supplementary material for: 68Ga-FAPI PET/CT for non-invasive characterization and activity assessment of ulcerative colitis and Crohn´s disease
Source: Eur J Nucl Med Mol Imaging. 2026 Jan 2;53(5):3258–70. doi: 10.1007/s00259-025-07686-1 (PMC13013222; doi:10.1007/s00259-025-07686-1)
Supplement: Supplementary file 1 — Supplementary file1 (DOCX 51 KB) [file 259_2025_7686_MOESM1_ESM.docx]

**^68^Ga-FAPI PET/CT for non-invasive characterization and activity assessment of ulcerative colitis and Crohn´s disease**

**SUPPLEMENTAL DATA**

Josefin Debus, Isabelle von Götze, Johannes Brandt, Robert Ehehalt, Anna-Maria Spektor, Philipp Mildenberger, Annika Gauss, Matthias Lang, Frederik M. Glatting, Mathias Schreckenberger, Rahul Kalla, Uwe Haberkorn and Manuel Röhrich

**Affiliations:**

1 Department of Nuclear Medicine, University Hospital Heidelberg, Germany.

2 Department of Nuclear Medicine, University Hospital Mainz, Germany.

3 Gastroenterology Outpatient Clinic Heidelberg, Germany.

4 Department of Gastroenterology, Hepatology and Infectious Diseases, University Hospital Heidelberg, Heidelberg, Germany.

5 Department of General, Visceral, and Thoracic Surgery, University Hospital Heidelberg, Germany.

6 Endokrinologikum Wiesbaden, Wiesbaden, Germany

7 Department of Radiation Oncology, University Medical Center Mannheim, Medical Faculty Mannheim, University of Heidelberg, Mannheim, Germany.

8 DKFZ-Hector Cancer Institute at the University Medical Center Mannheim, Mannheim, Germany.

9 Centre for Inflammation Research, Insititute for Regeneration and Repair, University of Edinburgh, Edinburgh, UK7 Clinical Cooperation Unit Nuclear Medicine.

10 German Cancer Research Center (DKFZ), Heidelberg, Germany

**First Author:** Josefin Debus
Im Neuenheimer Feld 400
69120 Heidelberg
Email: [josefin.debus@stud.uni-heidelberg.de](mailto:josefin.debus@stud.uni-heidelberg.de)

ORCID-ID: 0009-0006-5346-6965

**Corresponding Author:**

Manuel Röhrich
Langenbeckstraße 1
55131 Mainz
Telephone: 0049 6131 17 6516
Email: manuel.roehrich@unimedizin-mainz.de
ORCID-ID: 0000-0001-7609-243X

**Supplemental table 1**

| Gender | 27 male, 16 female |
| --- | --- |
| Age [years] | Range 5-71 (median 41) |
| Diagnosis [number of patients]: | |
| Sarcoma | **5** |
| Non-small cell lung cancer | **4** |
| Pancreatic cancer | **4** |
| Glioblastoma | **3** |
| Takayasu´s arteritis | **3** |
| Breast cancer | **2** |
| Thymoma | **2** |
| Adenoid cystic carcinoma | **2** |
| Cancer of unknown primary | **2** |
| Pancreatitis | **2** |
| Follicular lymphoma | **1** |
| Anaplastic astrozytoma | **1** |
| Paraganglioma | **1** |
| Neuroblastoma | **1** |
| Mucoepidermoid carcinoma | **1** |
| Medullary thyroid cancer | **1** |
| Melanoma | **1** |
| Serous low-grade ovarian cancer | **1** |
| Neuroendocrine carcinoma | **1** |
| Urothelial carcinoma | **1** |
| Intraductal papillary mucinous neoplasm | **1** |
| Interstitial lung disease | **1** |
| Dilated cardiomyopathy | **1** |

**Supplemental table 1:** Overview of diagnoses of the control group.

**Supplemental table 2**

| Patient number | Age [years] | Sex | Diagnosis | Time since diagnosis [years] | Mayo Endoscopic Score (MES)/  Ulcer presence | Disease activity | Duration of remission [years] | Current medical therapy |
| --- | --- | --- | --- | --- | --- | --- | --- | --- |
| 1 | 45 | m | CD | 3 | ulcers | active | - | none |
| 2 | 28 | m | CD | 8 | ulcers | active | - | Loperamide  Adalimumab (nac) |
| 3 | 18 | f | UC | 2 | MES 2 | active | - | Infliximab  Budesonide (nac) |
| 4 | 35 | f | CD | 19 | ulcers | active | - | Upadacitinib  Prednisolone (nac)  Risankizumab (nac) |
| 5 | 70 | m | UC | 10 | MES 0 | inactive | 3 | Infliximab |
| 6 | 60 | m | UC | 19 | MES 0 | inactive | 6 | Azathioprine |
| 7 | 65 | m | CD | 15 | ulcers | active | - | Budesonide |
| 8 | 38 | m | CD | 10 | ulcers | active | - | Infliximab |
| 9 | 30 | f | UC | 4 | MES 1 | inactive | <1 | Loperamide  Upadacitinib  Budesonide (nac)  Mesalazine (nac) |
| 10 | 32 | m | CD | 14 | ulcers | active | - | Ustekiunumab  Mesalazine  Budesonide (nac) |
| 11 | 57 | f | UC | 40 | MES 2 | active | - | Mesalazine |
| 12 | 33 | m | CD | 20 | ulcers | active | - | Mesalazine (nac)  Ustekinumab  (nac, instead of Adalimumab) |
| 13 | 30 | m | UC | 8 | MES 3 | active | - | Risankizumab  (nac, instead of Ustekinumab) |
| 14 | 69 | f | CD | 45 | ulcers | active | - | none |
| 15 | 21 | f | UC | 9 | MES 3 | active | - | Prednisolone  Budesonide  Mesalazine (nac)  Upadacitinib (nac) |
| 16 | 32 | m | UC | 14 | MES 3 | active | - | Ustekinumab  Budesonide  Loperamide  Upadacitinib (nac) |
| 17 | 36 | m | UC | 10 | MES 2 | active | - | Risankizumab (nac, instead of Adalimumab)  Prednisolone (nac)  Mesalazine (nac) |
| 18 | 63 | m | UC | 10 | MES 2 | active | - | Mesalazine  Budesonide  Tofacitinib |
| 19 | 57 | f | UC | 22 | MES 2 | active | - | Mesalazine  Budesonide  Prednisolone (nac) |
| 20 | 75 | m | UC | 14 | MES 3 | active | - | Mesalazine |
| 21 | 38 | f | CD | 8 | ulcers | active | - | Prednisolone  Risankizumab |
| 22 | 36 | m | CD | 16 | no ulcers | inactive | 3 | Adalimumab |
| 23 | 39 | f | UC | 21 | MES 3 | active | - | Mirikizumab (nac) |
| 24 | 38 | m | CD | 23 | ulcers | active | - | Risankizumab (nac, instead of Budesonide) |
| 25 | 30 | m | UC | 12 | MES 2 | active | - | Tofacitinib  Mesalazine (nac) |
| 26 | 39 | f | UC | 12 | MES 3 | active | - | Upadacitinib  Mesalazine  Budesonide  Prednisolone |
| 27 | 34 | f | CD | 14 | ulcers | active | - | Adalimumab |
| 28 | 21 | m | CD | 6 | no ulcers | inactive | 5 | Adalimumab |
| 29 | 58 | m | UC | 13 | MES 1 | inactive | 2 | Budesonide  Prednisolone |
| 30 | 50 | m | UC | 7 | MES 1 | inactive | 6 | Azathioprine  Mesalazine |
| 31 | 40 | f | CD | 25 | no ulcers | inactive | 1,5 | Ustekinumab |
| 32 | 47 | f | CD | 24 | ulcers | active | - | Risankizumab (nac)  Prednisolone (nac) |
| 33 | 50 | m | UC | 34 | MES 1 | inactive | 5 | Vedolizumab |
| 34 | 65 | m | CD | 20 | ulcers | active | - | none |
| 35 | 55 | m | CD | - | ulcers | active | - | Budesonide  Etoricoxib |
| 36 | 23 | m | UC | 1 | MES 3 | active | - | Budesonide  Mirikizumab (nac, instead of Infliximab) |
| 37 | 59 | m | UC | 28 | MES 0 |  | <1 | Ustekinumab  Mesalazin |
| 38 | 22 | f | CD | 6 | ulcers | active | - | Risankizumab  (nac, instead of Vedolizumab)  Mesalazine (nac) |
| 39 | 57 | m | UC | 14 | MES 1 | inactive | 4 | Infliximab  Mesalazine (nac) |
| 40 | 41 | f | CD | 9 | no ulcers | inactive | 4 | none |
| 41 | 25 | m | UC | 6 | MES 1 | inactive | 3,5 | Tofacitinib  Mesalazine |
| 42 | 46 | m | UC | 10 | MES 1 | inactive | <1 | Mesalazine  Budesonide |
| 43 | 46 | f | CD | 16 | ulcers | active | - | Prednisolone  Mesalazine |

**Supplemental table 2** Patientwise overview of diagnoses, colonoscopy results, disease activity and current medication of 43 inflammatory bowel disease (IBD) patients. Abbreviations: m: male, f: female, UC: ulcerative colitis, CD: Crohn’s disease, nac: new after colonoscopy and before ^68^Gallium- fibroblast activation protein inhibitor-based radiopharmaceuticals-46-positron emission tomography (^68^Ga-FAPI-46-PET).

**Supplemental table 3**

|  | Patients | Patients with IBD lesion | FAPI-positive GIT findings | FAPI-positive IBD lesions | Non-IBD-related FAPI-positive GIT findings |
| --- | --- | --- | --- | --- | --- |
| Inactive disease | 14 | 4 | 12 | 5 | 7 |
| Active disease | 29 | 29 | 68 | 61 | 7 |

**Supplemental table 3:** Distribution of inflammatory bowel disease (IBD)-related and non-IDB-related fibroblast activation protein inhibitor-based radiopharmaceuticals (FAPI)-positive gastrointestinal tract (GIT) findings in IBD patients with active and inactive disease.

**Supplemental table 4**

| Corticosteroid treatment | Patient number | SUVmean | SUVmax | Median SUVmean | Median SUVmax |
| --- | --- | --- | --- | --- | --- |
| Prednisolone (nac) | 4 | 3,1541 | 5,6517 | 2,7 | 4,4 |
|  |  |  |  |  |  |
|  | 17 | 3,0788 | 4,1880 |  |  |
|  |  |  |  |  |  |
|  | 19 | 2,3159 | 3,7012 |  |  |
|  |  | 2,3489 | 4,5938 |  |  |
|  |  | 3,7838 | 6,2261 |  |  |
|  |  |  |  |  |  |
|  | 32 | 1,8824 | 2,6989 |  |  |
|  |  |  |  |  |  |
| Budesonide (nac) | 3 | 2,0291 | 3,3860 | 2,3 | 4,8 |
|  | 9 | 3,1566 | 6,8163 |  |  |
|  | 10 | 2,4629 | 4,8346 |  |  |
|  |  |  |  |  |  |
|  |  | 1,9278 | 2,3739 |  |  |
|  |  |  |  |  |  |
|  |  | 2,6955 | 4,7278 |  |  |
|  |  | 1,5985 | 2,5750 |  |  |
|  |  | 2,1712 | 5,2157 |  |  |
|  |  | 5,1123 | 9,3211 |  |  |
|  |  |  |  |  |  |
| Prednisolone (bac) | 21 | 2,7937 | 4,0871 | 2,8 | 4,1 |
|  |  | 2,0647 | 2,9032 |  |  |
|  |  |  |  |  |  |
|  | 43 | 3,2379 | 5,2583 |  |  |
|  |  |  |  |  |  |
| Budesonide (bac) | 7 | 2,8416 | 6,5525 | 2,8 | 5,0 |
|  |  | 3,0020 | 7,3515 |  |  |
|  |  | 1,6352 | 3,7503 |  |  |
|  |  | 2,8071 | 4,9790 |  |  |
|  |  |  |  |  |  |
|  | 16 | 2,1508 | 4,2227 |  |  |
|  |  | 4,9271 | 9,6117 |  |  |
|  |  | 2,3199 | 3,0601 |  |  |
|  |  | 2,2575 | 3,5202 |  |  |
|  |  | 2,2022 | 4,5251 |  |  |
|  |  |  |  |  |  |
|  | 18 | 1,7366 | 2,4587 |  |  |
|  |  | 2,5594 | 3,5286 |  |  |
|  |  |  |  |  |  |
|  | 35 | 4,5222 | 7,5454 |  |  |
|  |  | 3,9115 | 6,6949 |  |  |
|  |  |  |  |  |  |
|  | 36 | 3,8609 | 7,1153 |  |  |
|  |  | 4,2614 | 6,3325 |  |  |
|  |  |  |  |  |  |
|  | 42 | no lesion | no lesion |  |  |
|  |  |  |  |  |  |
| Prednisolone & Budesonide (bac) | 15 | 3,7580 | 7,1675 | 3,8 | 5,8 |
|  |  |  |  |  |  |
|  | 26 | 3,8655 | 5,6217 |  |  |
|  |  | 4,5685 | 6,0085 |  |  |
|  |  |  |  |  |  |
|  | 29 | 2,1257 | 2,9746 |  |  |
|  |  |  |  |  |  |
| Any corticosteroid treatment |  |  |  | 2,7 | 4,8 |
|  |  |  |  |  |  |
| No corticosteroid treatment | 1 | 1,9214 | 3,2094 | 2,7 | 4,3 |
|  |  | 2,5065 | 8,6795 |  |  |
|  |  |  |  |  |  |
|  | 2 | 2,7539 | 6,6690 |  |  |
|  |  | 3,0213 | 5,8759 |  |  |
|  |  |  |  |  |  |
|  | 5 | 1,1979 | 2,8245 |  |  |
|  |  | 0,9990 | 2,0645 |  |  |
|  |  |  |  |  |  |
|  | 6 | no lesion | no lesion |  |  |
|  |  |  |  |  |  |
|  | 8 | 4,4740 | 8,0266 |  |  |
|  |  |  |  |  |  |
|  | 11 | 1,2351 | 1,9888 |  |  |
|  |  | 1,5393 | 2,2674 |  |  |
|  |  | 2,3483 | 4,0737 |  |  |
|  |  | 1,1690 | 2,2302 |  |  |
|  |  | 1,9061 | 3,1094 |  |  |
|  |  |  |  |  |  |
|  | 12 | 2,7014 | 4,5508 |  |  |
|  |  | 6,0244 | 12,7198 |  |  |
|  |  |  |  |  |  |
|  | 13 | 2,8009 | 5,9319 |  |  |
|  |  |  |  |  |  |
|  | 14 | 2,6060 | 5,8762 |  |  |
|  |  |  |  |  |  |
|  | 20 | 2,9095 | 4,2427 |  |  |
|  |  | 4,9043 | 9,7289 |  |  |
|  |  | 2,9428 | 4,1594 |  |  |
|  |  |  |  |  |  |
|  | 22 | no lesion | no lesion |  |  |
|  |  |  |  |  |  |
|  | 23 | 3,6045 | 4,3568 |  |  |
|  |  | 4,0104 | 5,5091 |  |  |
|  |  |  |  |  |  |
|  | 24 | 9,1485 | 14,6682 |  |  |
|  |  |  |  |  |  |
|  | 25 | 3,2630 | 4,8823 |  |  |
|  |  |  |  |  |  |
|  | 27 | 3,0125 | 4,2941 |  |  |
|  |  | 3,1376 | 4,8636 |  |  |
|  |  |  |  |  |  |
|  | 28 | no lesion | no lesion |  |  |
|  |  |  |  |  |  |
|  | 30 | no lesion | no lesion |  |  |
|  |  |  |  |  |  |
|  | 31 | 2,5343 | 3,2907 |  |  |
|  |  |  |  |  |  |
|  | 33 | no lesion | no lesion |  |  |
|  |  |  |  |  |  |
|  | 34 | 1,8037 | 2,8459 |  |  |
|  |  | 1,9628 | 2,9095 |  |  |
|  |  |  |  |  |  |
|  | 37 | no lesion | no lesion |  |  |
|  |  |  |  |  |  |
|  | 38 | 2,7441 | 4,2606 |  |  |
|  |  | 3,1859 | 6,0029 |  |  |
|  |  |  |  |  |  |
|  | 39 | no lesion | no lesion |  |  |
|  |  |  |  |  |  |
|  | 40 | no lesion | no lesion |  |  |
|  |  |  |  |  |  |
|  | 41 | no lesion | no lesion |  |  |

**Supplemental table 4:** ^68^Gallium- fibroblast activation protein inhibitor-based radiopharmaceuticals-46 (^68^Ga-FAPI-46)-uptake in inflammatory bowel disease (IBD) lesions of patients with and without corticosteroid medication. Abbreviations: nac: new after colonoscopy and before ^68^Gallium-FAPI- positron emission tomography (PET), bac (before and after colonoscopy).
